# Nonlinear Viscoelasticity of and Structural Modulation in Guar Gum-Enhanced Triple-Network Hydrogels

**DOI:** 10.3390/polym17050597

**Published:** 2025-02-24

**Authors:** Yi Luo, Werner Pauer, Gerrit A. Luinstra

**Affiliations:** Institut für Technische und Makromolekulare Chemie, Universität Hamburg, 20146 Hamburg, Germany; yi.luo@chemie.uni-hamburg.de (Y.L.); werner.pauer@uni-hamburg.de (W.P.)

**Keywords:** guar gum, large-amplitude oscillatory shear (LAOS), crystallinity

## Abstract

The effect of the presence of guar gum (0–0.75 wt%) in a thermo-responsive triple-network (TN) PVA/TA/PVA-MA-g-PNIPAAm hydrogel (PVA: polyvinyl alcohol; MA: methacrylate, PNIPAAm: poly-N-isopropyl acryl amide; TA: tannic acid) with respect to the structural, mechanical, and viscoelastic properties was mapped. A comprehensive analysis, using large-amplitude oscillatory shear (LAOS), SEM imaging, XRD, and mechanical analysis revealed that guar enhances hydrogel crystallinity (up to 30% at 0.75 wt%), which goes along with a strain hardening. The hydrogel achieved superior mechanical performance at a gum concentration of 0.5 wt% with a 40% increase in shear-thickening, an enhanced strain tolerance in nonlinear regimes, and a good mechanical robustness (maximum elongation to break of 500% and stress of 620 kPa). The hydrogel with 0.5 wt% guar exhibited also a good thermal response (equilibrium swelling ratio changed from 8.4 at 5 °C to 2.5 at 50 °C) and an excellent thermal cycling dimensional stability. Higher guar concentrations reduce structural resilience, leading to brittle hydrogels with lower extensibility and viscoelastic stability.

## 1. Introduction

Guar gum, a natural polysaccharide extracted from the endosperm of the guar bean (Cyamopsis tetragonoloba), is renowned for its exceptional thickening, stabilizing, and water-retaining properties. It dictates the material’s mechanical strength and swelling capacity, resulting in a wide range of applications such as drug delivery [1,2,3], tissue engineering [4], and regenerative medicine [5,6]. Its molecular structure consists of a linear backbone of β-1,4-linked D-manno pyranosyl units with α-1,6-linked D-galactopyranosyl side chains, conferring upon it high water solubility and the ability to form viscous solutions even at low concentrations [7,8,9]. The mechanism underlying guar gum’s efficacy in hydrogel formation involves its capacity to create entangled networks—also comprising, most likely, hydrogen-bonding-based “hyperentanglements”—upon hydration, significantly enhancing the viscoelastic property profile [10,11,12]. Such networks are crucial for maintaining the structural integrity of hydrogels while encapsulating large volumes of water, a property that can be explored for its potential in creating responsive and durable hydrogel matrices. Despite these advantages, there appears to be a limited understanding of how guar gum affects the physical behavior of hydrogels, especially concerning its nonlinear viscoelastic behavior at large deformations. The rheological properties of guar gum-based hydrogels, such as their shear thinning behavior and recoverable viscoelasticity, allow for the adjustment of the hydrogel’s mechanical properties, including its stiffness and elasticity, which are critical for the hydrogel’s performance in diverse environments. Guar gum’s inherent qualities enable the crafting of hydrogels with specific functionalities, highlighting the importance of guar in the evolution of hydrogel technology for sophisticated and multifunctional material systems [11,13,14]. Guar gum has been successfully used to form single-network hydrogels, but its role in complex, triple-network (TN) architectures appears largely unexplored. This study, therefore, sought to bridge this knowledge gap by incorporating guar gum into a TN hydrogel system and elucidating its impact on structural integrity, viscoelastic behavior, and mechanical robustness.

The motivation for developing this hydrogel stemmed from previous work on a thermo-responsive, self-healing PVA/TA/PVA-MA-g-PNIPAAm system, which demonstrated promising mechanical properties and enhanced thermal stability [15]. Tannic acid was a major driver for improving the mechanics and self-healing of the PVA/PVA-MA-g-PNIPAAm basic gel [16]. However, a key limitation was observed: during repeated swelling and deswelling cycles, the gel exhibited progressive dimensional shrinkage, ultimately compromising its long-term stability. It is anticipated that the inclusion of high-molecular-weight guar gum, with its multiple entanglements and its dynamic interactions within the amorphous phase, could counteract the undesired contraction and thus improve dimensional stability in multiple swelling–deswelling cycles.

Insights into the structure across varying concentrations of guar of the resulting hydrogels were elaborated using a combination of mechanical and rheological methods, with an important contribution from large-amplitude oscillatory shear (LAOS) rheometry. The latter is predestinated for reaching insights into the behavior of a material in real applications. Large-amplitude oscillatory shear (LAOS) is an advanced rheological technique that is crucial for probing the complex viscoelastic properties of materials subjected to larger, i.e., nonlinear, deformations, which are more relevant to common applications [7,17]. The technique enables the identification of strain-dependent moduli and the deconstruction of stress responses into nonlinear components, providing a delicate understanding of the material behavior [18,19,20]. It was envisioned that LAOS might be useful for an investigation into the hydrogels’ structural integrity and functional limits under stress conditions, and also those imposed at swelling and deswelling operations [21,22,23,24]. The impact of guar gum on the hydrogel’s nonlinear viscoelastic behavior, a critical factor in ensuring robustness under mechanical stress, was mapped using the LAOS framework.

The assessment of nonlinear viscoelastic properties was performed at room temperature using guar gum’s capacity to attain maximum viscosity at “cold water” conditions. This property is attributed to the hydroxyl groups along the guar chains, which engage in hydrogen bonding within an aqueous and hydroxyl OH-containing environment. This structural arrangement will interact with the triple network system and may add dimensional rigidity, possibly through the longer relaxation times of the “hyperentangled” guar chains [25]. Such networks could lead to the development of a more complex mechanical structure within the hydrogel. This work provides a comprehensive understanding of the interplay between guar gum, PVA crystallization, and TN hydrogel architecture. The findings hold potential for guiding the design of next-generation hydrogels with enhanced dimensional stability, tunable mechanical responses, and improved durability for biomedical and smart material applications.

## 2. Materials and Methods

### 2.1. Materials

The hydrogel synthesis was carried out with the following chemicals. Poly(vinyl alcohol) (PVA), with a molecular weight range of 85,000 to 124,000, hydroquinone, and N, N’-methylene bisacrylamide (MBA), was procured from Sigma-Aldrich, Taufkirchen, Germany. Lithium phenyl-2,4,6-trimethyl-benzolphosphonate (TPO-Li), triethyl amine, tannic acid, and guar (guar gum) were also procured from Sigma-Aldrich. N-isopropyl acrylamide (NIPAAM) was obtained from Acros Organics, Darmstadt, Germany, and methacrylic acid came from Merck KGaA, Darmstadt, Germany. The experiments utilized demineralized water with low conductivity, indicative of high purity, from laboratory water systems.

### 2.2. Hydrogel Preparation

PVA-MA was prepared using a previously established method. A quantity of 15 g of polyvinyl alcohol (PVA) was dissolved in 135 mL of deionized water in a round-bottom flask with a condenser and stirred at 90 °C for 1 h. After cooling, 30 mg of hydroquinone, 20 mL of methacrylic acid (MA), and 7.5 mL of 0.5 M hydrochloric acid were added, followed by stirring at 60 °C for 12 h to complete the esterification. The solution was neutralized with 0.5 mL of triethyl amine, then diluted to 1.5 L with water and mixed with 1.5 L of acetone to precipitate the polymer, which was filtered, washed, and dried at 60 °C under 1000 mbar vacuum.

A hydrogel blend was created by dissolving 5 wt% PVA and 5 wt% PVA-MA in deionized water, heated, and stirred at 90 °C for an hour. Tannic acid (1 g dissolved in 1 mL water at 50 °C) was subsequently incorporated into the PVA/PVA-MA mixture with continuous stirring. Following the incorporation of tannic acid, the mixture was allowed to cool to ambient temperature, after which 20 wt% N-isopropylacrylamide (NIPAAM) and different contents of guar were added; stirring was maintained until a homogenous mixture was obtained. Polymerization was initiated by adding 0.1 wt% TPO-Li and 0.2 wt% MBA, then degassed via ultrasonic treatment for over 30 min.

The mixture was transferred into various shaped molds made from polylactic acid (PLA) through the Fused Deposition Method (FDM) using an Ultimaker 2 3D printer (Ultimaker B.V., Framingham, MA, USA). The filled molds were exposed to ultraviolet (UV) light with a maximum intensity of 1100 mW/cm^2^ and a wavelength of 365 nm, sourced from LED Cube 100 IC chamber (Dr. Hönle AG, Nicolaus-otto-Str 2, Gilching, Germany), under the set of 10% LED power for 600 s. After UV exposure, the hydrogels were de-molded, immersed in deionized water for 24 h to remove impurities, and subjected to a freeze–thaw cycle (−32 °C for 12 h, thawed at 25 °C for 2 h) for final hydrogel preparation and preserved in deionized water for further use.

### 2.3. Characterization

#### 2.3.1. Fourier-Transform Infrared (FTIR) Spectroscopy

For spectral analysis, the Attenuated Total Reflectance–Fourier-Transform Infrared (ATR-FTIR) spectra of the samples were obtained by Vertex 70 instrument (BRUKER OPTIK GmbH, Ettlingen, Germany), over a spectral range spanning from 4000 to 400 cm^−1^, with a resolution of 2 cm^−1^, through 32 scans.

#### 2.3.2. Scanning Electron Microscopy (SEM)

The samples were lyophilized for investigating the morphologies., The specimens were initially cooled with liquid nitrogen for 10 min before being transferred to a freeze dryer (Gamma 2-16 LSCplus, Osterode am Harz, Germany) where they underwent lyophilization for 48 h. A sputtering process was applied to coat each sample with a thin Pt layer. Subsequently, the examination of surface morphology was conducted utilizing a LEO 1525 Field Emission Scanning Electron Microscope (LEO Electron Microscopy Inc., One Zeiss Drive, Thornwood, NY, USA) operating at an accelerating voltage of 5 kV.

#### 2.3.3. X-Ray Diffraction (XRD)

The investigation into the crystalline structure of the hydrogel samples was performed utilizing X-ray diffraction (XRD) analysis. The samples were firstly dried within a vacuum oven at a temperature of 80 °C throughout the night to ensure the thorough elimination of any moisture content. These dried samples were then mechanically processed into a fine powder form. This analysis was conducted at room temperature using a Pulverdiffraktometer Panalytical MPD X’Pert Pro (STOE & Cie GmbH, Darmstadt, Germany), which was set with a generator output of 45 kV and a current of 40 mA. The diffraction patterns were scanned over a 2θ range extending from 5° to 100°, with a scanning step size of 0.02° per step.

#### 2.3.4. Differential Scanning Calorimetry (DSC)

Mettler-Toledo dsc 1 star system (Mettler Toledo GmbH, Gießen, Germany) was employed for assessing the crystallinity degrees and crystal size distributions of the hydrogel. Each sample, typically weighing between 5 to 10 mg and partially dried, was placed in an aluminum pan. The sample was then heated at a rate of 5 °C/min, ranging from 30 °C to 300 °C. To ensure a consistent and stable thermal condition, a nitrogen flow was directed through the chamber holding the sample.

#### 2.3.5. Tensile Strength

Tensile testing was conducted using a Zwick Z2.5 universal testing machine (Zwick/Roell, Ulm, Germany) in accordance with ASTM D412 standards. Specimens with a dumbbell shape (4 mm in width, 2 mm in thickness, and 25 mm in length) were fabricated using a specifically designed 3D printed mold. They were secured at both ends within the testing machine and elongated at a uniform rate of 5 mm/min until fracture. The stress and strain at the breaking point were documented through the stress–strain curves.

#### 2.3.6. Swelling Properties

The investigation into the hydrophilicity of the hydrogel utilized a swelling equilibrium experiment conducted over a temperature range of 6 °C to 50 °C. In this context, the equilibrium swelling ratio (ESR), calculated as (Ws/Wd * 100%), represented the ratio of the hydrogel’s weight in its water-swollen state (Ws) to its weight in the dehydrated state (Wd). Hydrogel specimens were crafted using a 3D-printed circular mold of 3 cm in diameter and subsequently submerged in deionized water for 12 h to achieve equilibrium. The specimens were then wiped to remove any residual surface water before being weighed. The drying phase entailed placing the samples in a vacuum dryer overnight to ensure thorough dehydration, after which the dehydrated samples’ weights were recorded.

The swelling dynamics were visually monitored as the temperature varied from ambient to above the lower critical solution temperature (LCST). A series of circular specimens were repeatedly immersed in the water bath in low (room temperature) and high (above LCST) temperatures, with each process lasting for 30 min, during which a camera captured the volumetric alterations. The assessment of these dimensional variations was conducted utilizing Imagine-Pro Plus 6.0 software (Media Cybernetics, Rockville, MD, USA). For quantifying the swelling ratios, the length-normalized swelling degrees, Q_L_, were determined by the formula $Q_{L}=\frac{L_{S}}{L_{Q}}*100\%$, wherein L_S_ represents the length of the hydrogel post swelling and L_Q_ denotes the length of the hydrogel at equilibrium.

#### 2.3.7. Rheological Measurements

Rheological measurements were carried out utilizing a DHR-2 combined motor-transducer rheometer (TA Instruments, New Castle, DE, USA) equipped with a plate of 20 mm diameter and a 1000 μm measuring gap. The evaluation of the self-healing capabilities of the prepared hydrogels was conducted through dynamic rheological testing. These tests involved subjecting the hydrogel samples to cycles of amplitude oscillatory strains, ranging from small strains (γ = 1%) to large strains (γ = 250%), with each strain level being applied for a duration of 100 s. Throughout this procedure, the storage modulus and loss modulus were continuously monitored and recorded, maintaining a constant frequency of 1 Hz at ambient temperature.

The large-amplitude oscillatory shear (LAOS) experiments were conducted across a frequency spectrum ranging from 1 to 31.6 rad/s, employing the rheometer’s active deformation control in continuous oscillation mode. The implementation of advanced features in the deformation control feedback loop and the precise setting of the motor mode in stress-controlled rheometers facilitated a notable congruence with the nonlinear behaviors observed in strain-controlled rheometers, with the motor mode being adjusted to a stiff configuration.

The processing of the collected data was achieved using the MITlaos software (version 2.1), a creation of Ewoldt, Winter, and McKinley in 2007 [26]. This involved the application of at least five strain cycles, preceded by two conditioning cycles, for the calculation of Chebyshev and Fourier coefficients. A discrete Fourier Transform analysis was utilized to identify the most significant harmonic within the raw data, which then served as the basis for reconstructing a stress signal with minimized noise. This reconstructed signal was subsequently analyzed further.

## 3. Results and Discussion

### 3.1. Hydrogel Formation

**Figure 1:**
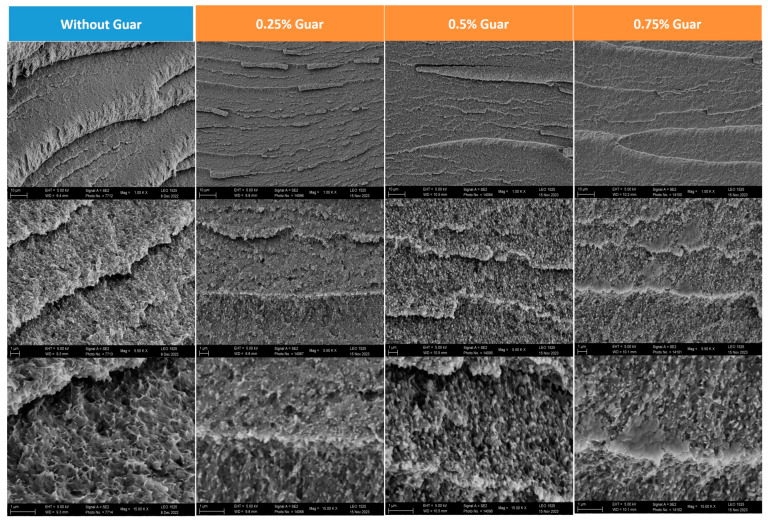
SEM images of the hydrogels with guar gum (magnifications of 1.00, 5.50, and 15.00 K×).

Hydrogels were prepared from an aqueous solution of PVA (5 wt%) and PVA-MA (5 wt%), NIPAAm (20 wt%), BIS (0.1 wt%), a crosslinker (0.2 wt%), tannic acid (0.5 wt%), guar gum (0–0.75 wt%), and a photoinitiator (phenyl-2,4,6-trimethyl benzoyl phosphinate) [27]. Acrylic polymerization was induced by UV light after placing the solution in a mold and degassing. The triple network of the PVA/TA/PVA-MA-g-PNIPAAm hydrogel had been recently introduced as a thermal-responsive hydrogel [15]. Tannic acid (TA) contributes by the addition of self-healing properties and mechanical strength by forming dynamic hydrogen bonds; the best proportion of 0.5 wt% was applied here. The FT-IR spectrum (Figure S1) of the precursor solution featured a loss of the acryl amide stretch vibrations at 1650 cm^−1^ and 1610 cm^−1^. The photoinduction of the polymerization gave rise to a new C=O vibration at 1625 cm^−1^, illustrating the successful polymerization in the presence of guar. Concomitantly, the aliphatic CH stretch vibration near 2940 cm^−1^ became more prominent and the C=C-H bend vibrations near 990 cm^−1^ disappeared. The final hydrogels were obtained after subjecting the initial resin to a freeze–thaw cycle (F–T cycle) to induce PVA crystallization. The resulting hydrogel specimens were then kept in a refrigerator (4 °C) for subsequent characterization. The hydrogels manifested themselves after drying in SEM as three-dimensional foams (Figure 1).

**Figure 2:**
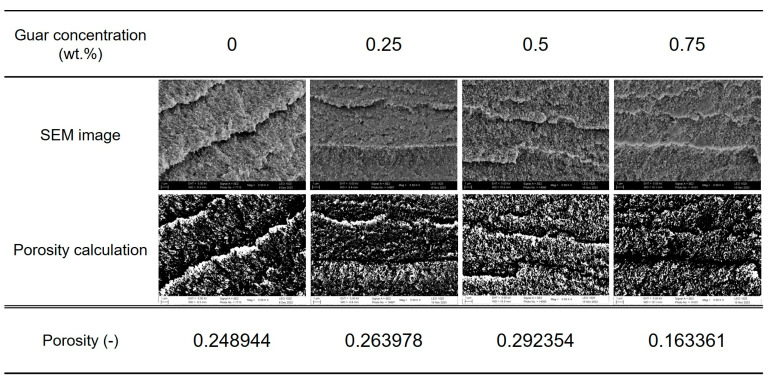
Porosity (Φ) of hydrogels with different contents of guar, calculated as VV/VT, where VV is the volume of void-space (such as fluids) and VT is the total or bulk volume of material.

A correlation existed between the XRD pattern, the DSC thermal features, and the images obtained from the SEM (Table 1; Figure 1). Only subtle differences appeared in the imaging at first sight. A closer inspection hinted at a structure with ever thinner and more tightly packed layers along with the amount of guar (Figure 2). An increase in guar concentration correlated with a reduction in the sizes of macroscopic pores, implying that guar subtly influences PVA crystal growth: the amount of guar was so low that it should not have directly changed the structure.

The presence of polyvinyl alcohol (PVA) crystallites, the physical crosslinking junctions created through freeze–thaw (F–T) processes, was evidenced by characteristic peaks at diffraction angles of 8.8°, 19.8°, and 41.1° (Figure S2). Analysis revealed a consistent pattern where the XRD peaks indicated an increase in overall crystallinity with the guar concentration (Table 1, Figure S2). The width at half maximum (FWHM) decreased somewhat with the guar content, indicative of more ordered and or larger crystals, of which the latter was not reflected in the melting behavior. The melting range was basically the same; the maximum seemed to randomly change somewhat. The crystallinity (by DSC), however, increased noticeably with the guar content and was generally much higher than in the gel without guar. Guar gum thus seems a directing factor in the crystallization of PVA, even at very low concentrations.

Higher contents of guar, like in a gel with 1 wt%, lead to brittle products that could not be handled without a loss of integrity. The gel with 1 wt% guar before crystallization was sticky and gluey. This was indicative of an interference of the guar with the polymerization process. Guar contains weak C-H bonds that may act as chain terminators. The resulting radicals on the guar chain may then react with acrylic monomer to give a functionalized guar or may undergo a decomposition.

### 3.2. Mechanical Properties

**Figure 3:**
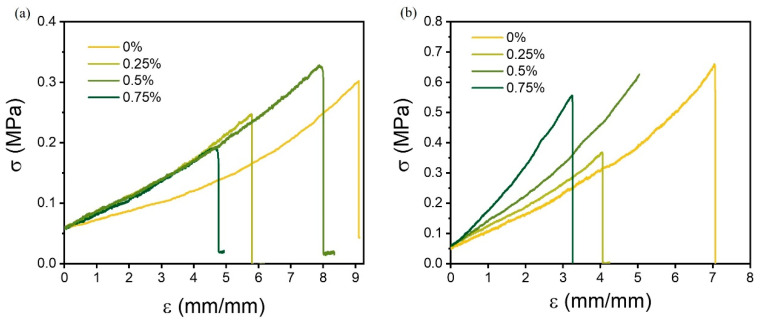
Fracture tensile curves of the guar hydrogels before (**a**) and after crystallization (**b**) in an F–T operation.

The stress–strain behaviors of the hydrogels with different guar content directly after photopolymerization (prior to PVA crystallization) showed similar slopes (Figure 3a). This indicated that a similar network was resisting extension, which was a composite of the chemical network from the acrylate crosslinking with BIS and the various hydrogen bonding interactions. The maximum elongation before breakage was increasing with the guar content to about 0.5 wt% at 800%. Higher guar contents resulted in a failure at lower extension. The delocalization of the imposed stress was hampered at the higher guar concentration (Table S1). A possible explanation was the higher number of bonds between guar chains (the hyperentanglements), but, more likely, radical abstraction reactions at the guar backbone had led to the presence of more dangling chain ends in the acrylate network, impeding the ability to delocalize stress [28,29]. The latter view was compatible with the sticky appearance after the photopolymerization of the monomer-containing solution at guar concentrations of 1 wt% or higher.

These observations aligned with the crystallinity data from XRD and DSC (Table 1), which confirmed that a higher guar content promoted PVA crystallization. However, excessive crystallization at 0.75 wt% resulted in network rigidity, reducing elongation capabilities. The crystallization of PVA in the F–T procedure led to the usual increase in the stiffness of the gels. A trend of a higher Emod with a higher content of guar, concomitantly with a higher crystallinity, was general, but the ductility was not. The high ductility of the basis gel (no guar gum) was related to its lower crystallinity, which provided longer amorphous mobile links. The addition of the smallest amount of 0.25 wt% guar in this study increased the crystallinity, resulting in an incremental increase in stiffness and a larger impact on ductility, leading to a break at low stress and extension. The tensile properties of the hydrogel with 0.5 wt% of guar with only a 2% higher crystallinity (i.e., a 10% increase) showed the highest stress of 620 kPa. This higher attainable stress followed also from the much higher elongation of 500%. In contrast, the gel prepared with 0.75 wt% of guar was less ductile and broke already at less than 350% elongation, closer to the behavior of the gel with 0.25 wt% of guar gum.

The presence of guar thus at least has two different effects on the mechanical property profile of the gel. One is related to the crystallization: its presence leads to a higher crystalline content with smaller crystals. This behavior is related to the ability of guar gum to inhibit ice crystal growth by slowing down the mass transfer across the solid–liquid interface. The PVA solution will undercool to a larger extent and more nucleation will take place to give smaller crystallites and a higher overall crystallinity. Also, the number of tie molecules between the crystals should increase that way [30]. At the same time, the BIS-MA network formation is hampered by terminations, tentatively involving the weak C-H bonds of guar (possibly also leading to guar chain breakage), which was evident from the virgin gels before the F–T procedure (Figure 3a) [31]. The overall effect of guar seems to be the balance between a less continuous PVA-MA BIS network and a higher crystallinity in the PVA part, possibly attenuated by a further connectivity on account of guar’s high molecular weight. A “sweet spot” with respect to the material property profile is the gel with 0.5 wt% of guar.

### 3.3. Rheological Properties

#### 3.3.1. Strain Sweep

Further insights into the network at various strains are of interest, also with the objective to at least reach an impression of the importance of mechanical stress for the swelling dynamics. The gels undergo an appreciable contraction/expansion during swelling after equilibration, which could make them potentially useful as smart components in a valve (vide infra) [32]. Also, the hypothesis of guar acting as an effective chain transfer agent needs attention; the resulting network should show a corresponding dependence.

The storage modulus (G′) and the loss modulus (G″) at a low amplitude and frequency of 1 Hz were about an order of magnitude apart (G′ >> G″), as was expected for a genuine hydrogel [33]. Increasing the strain led to a decrease in G′, signaling a compromise of the structural integrity. This transition to non-Newtonian responses was coarsely delineated by two pivotal strains: the initial critical strain (γ_c_), from which point G′ became sensitive to deformation, and a subsequent yield strain (γ_y_), at which G′ equaled the loss modulus (Table 2).

The inclusion of even the smallest amount of guar was found to decrease the initial critical strain, indicating that the gel indeed was more crosslinked with smaller arcs between the crystallites. Possibly, the entanglements of the guar, which may have had a longer time scale than 1 Hz, may also have reduced the critical strain [34]. The higher strains thus led to irreversible changes in the gel. A complete breakdown was not observed; possibly, the presence of TA giving it a self-repairing quality was important in securing a certain robustness.

The initial critical strain increased again when the guar content was raised from 0.25 to 0.5 wt%, a clear sign of guar involvement—either directly or by a change in crystal morphology—in the mechanics of the gel. The higher critical strain was indicative of a network that was more flexible. Termination reactions in the radical polymerization process, caused by C-H abstraction on the guar backbone, would result in a reduced crosslinking density within the NIPAAm-based network. The generation of radical entities on guar would contribute to a reduction in guar’s molecular weight during polymerization. The chain transfer reactions may have led to an increase in the fraction of dangling chain ends, ultimately affecting the elasticity and mechanical stability of the hydrogel [35]. The lower molecular weight of the guar and the resulting lesser connected PVA network allowed for a larger elastic deformation despite the finer morphology of the PVA crystallites. A further increase in guar content then prevented the formation of a substantial crosslinked network and a loss of structure occurred at much lower deformations. This was compatible with the SEM images and the morphology, which indicated a substantial interference between the PVA network formation by guar, changing the nature of the gel.

The loss moduli G″ of the respective gels were in accordance with the guar interfering with PVA network formation. The G″ of the gels with guar were higher than the basic gel at low strain in the linear viscoelastic region, showing the general influence of guar in the system at deformation. The loss modulus had a notable peak as a function of the extension. The initial increase was interpreted to result from the higher friction among the segments in the extended network at the higher rates according to Newton‘s law of viscosity. The subsequent decline at still higher amplitudes was indicative of network disintegration.

The maximum in G″ was found at lower values of amplitudes for the gels with guar, again illustrating the restriction of movements in the resulting gels with a concomitant support of integrity. Again, the gel with 0.5 wt% of guar had the superior combination of properties: it had the most significant increase in G″ (Figure 3). It had a larger amplitude of deformation at maximum in agreement with the interpretation of a lesser dense NIPAAm network (Table 2, Figure S3). At the same time, the gel with 0.75 wt% of guar reached the lowest maximum of G″ at the lowest amplitude of the guar-based gels. A drastic drop of γ_y_ (from 40% to 10%) was observed at 0.75 wt% guar, aligning with mechanical property trends that highlighted the destabilization of the system with higher amounts of guar (Table 2).

The square of the average molecular weight between effective crosslinks ${\bar{M_{e}}}^{2}$ (from $\bar{M_{e}}=\frac{\rho RT}{G\prime }$) scaled, as expected for a rubbery network, linearly with the strain at break (λ_max_) (Table S2, Figure S4) for the guar-containing hydrogels [36]. This correlation suggested that guar enhanced the homogeneity of the crosslinked network by promoting hydrogen bonding and influencing PVA crystallization, which aligned with the theory that larger $\bar{M_{e}}$ values corresponded to increased extensibility due to reduced crosslink density and higher chain mobility [37]. In contrast, the deviation of the 0 wt% guar sample from this linear trend indicated that the hydrogel without guar exhibited fewer transient interactions and a less entangled network, leading to irregular stress distribution and a lower extensibility. These findings corroborated the interpretation that emphasized the significance of guar in enhancing PVA crystallization and molecular interactions, which established a more predictable relationship between network structure and mechanical behavior.

#### 3.3.2. Advanced Structure Analysis by LAOS

**Figure 4:**
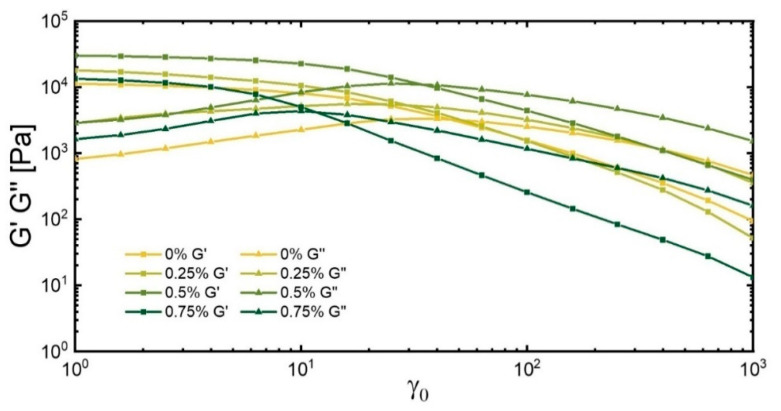
Storage modulus G′(γ_0_) and loss modulus G″(γ_0_) across different concentrations of guar hydrogels.

The rheological behavior of viscoelastic materials, including hydrogels, can effectively be characterized by analyzing their response to a periodic stress. The stress response remains sinusoidal in small oscillatory shear (SAOS) experiments, allowing the storage modulus (G′) and loss modulus (G″) to maintain their meaning in terms of viscoelastic interpretations (like in Maxwell elements). This response σ(t) in the relevant larger strains of application is bound to be nonlinear. As the strain amplitude (γ_0_) increases and the system enters the nonlinear regime, the stress output deviates from a sinusoidal pattern (Figure S5). In this nonlinear regime, the relaxation modulus (G(t)) is influenced by both the strain amplitude and the frequency of oscillation, rendering the assumption of small strain gradients invalid [38]. Hydrogels typically transition from elastic-dominant to plastic and yielding behavior as the strain amplitude increases in LAOS [23]. In complex hydrogel systems, various mechanisms have been identified mainly according to shear-induced temporary structural reorganization and changes in molecular interactions (Figure 4) [21,39].

**Figure 5:**
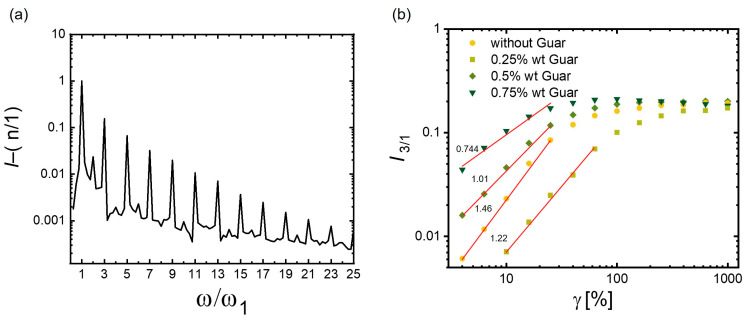
(**a**) Fourier-Transform rheology spectrum, displaying relative intensity (I_n/1_) versus normalized harmonic frequency (ω/ω_1_) for hydrogel with 0.5 wt% of guar, under an excitation angular frequency of 1 rad/s at 1000% amplitude and (**b**) relative intensity of the third harmonic (I_3/1_) and the slopes at MAOS.

An approach known as F–T rheology decomposes the response into a series of sinusoidal and cosinusoidal components [17]. The in-phase sinusoidal part of the response is related to the elastic properties with Fourier coefficients $G\prime_{n}$ (along Hooke’s law) and the out-of-phase answer to the viscous properties with coefficients $G{\prime \prime }_{n}$. The stress response in mathematical terms thus is formulated as $\sigma \;\left(t\right)=\gamma_{0}\underset{n=odd}{\sum }\left\{G_{n}^{\prime }\right.\left(\omega ,{\;\gamma }_{0}\right)\sin n\omega t+G_{n}^{\prime \prime }\left(\omega ,{\;\gamma }_{0}\right)\left.\cos n\omega t\right\}$. The Fourier-Transform (FT) rheological analysis of the hydrogels in this study revealed a pronounced nonlinear flow. The gel based on 0.5 wt% of guar, for example, showed thirteen discernible harmonics at a deformation of γ_0_ of 1000% at a frequency of 1 rad/s (Figure 5a) that refined the stress response.

The degree of nonlinearity within the response could be quantified by examining the ratio of the intensity of the third harmonic to the fundamental excitation, I_3_/I_1_ [17]. This ratio exhibited an exponential relationship with the strain amplitude, γ_0_, particularly within the medium strain amplitude range (1% < γ_0_ < 100%), where the magnitude of the I_3_/I_1_ slopes provided a perspective on the polymer structure. For example, studies had shown that for linear polydisperse polypropylene (PP), this slope was 2, but for branched PP, it was lower at 1.64 [40]. A lower value was associated with a strain hardening on account of restricted chain segment movements imposed by the entanglements.

The magnitude of I_3_/I_1_ slopes of the different gels in this study was quite sensitive to the amount of guar: as the guar content increased from 0.0 to 0.75 wt%, the I_3_/I_1_ slope decreased from 1.46 to 0.744 in the medium range of strains (MAOS: medium amplitude oscillatory strain; Figure 5). The gradual reduction in I_3_/I_1_ slopes was consistent with the progressive transition from a more open network to one with smaller arcs between the crosslinks. It emphasized the hydrogel’s enhanced strain hardening effect as the guar content strengthened the network by forming additional transient crosslinks and interacting with PVA and tannic acid through hydrogen bonding and hydrophobic interactions next to the increased crystallization (rate) of PVA. All these crosslinks acted as strain-tolerant energy dissipators, enabling the hydrogel to withstand higher deformations. The LAOS analysis thus amplified the impression of the constitution of the gels as a function of the guar content from SEM and XRD analysis and clearly showed the implications for the mechanical profile.

#### 3.3.3. Stress Decomposition and Lissajous–Bowditch Curves

A more visual depiction of the nonlinear response was reached using the orthogonal stress decomposition (SD) method [17]. The total stress response in that analysis was described in terms of Chebyshev polynomials, also allowing us to separate elastic, strain-dependent and viscous, strain-rate-dependent components [26,41]. Formally, the relationship between the total stress response and elastic and viscous contributions was represented as $\sigma \left(t;\omega ,\gamma_{0}\right)=\gamma_{0}\underset{n,odd}{\sum }e_{n}\left(\omega ,\gamma_{0}\right)T_{n}\left(x\right)+{\dot{\gamma }}_{0}\underset{n,\;odd}{\sum }v_{n}\left(\omega ,\gamma_{0}\right)T_{n}\left(y\right)$, wherein *e_n_* and *v_n_* were the coefficients of the fundamental Chebyshev polynomials $T_{n}$. Using this basis (instead of the harmonics of the FT-analysis), nonlinear viscoelastic moduli G′_M_ and G′_L_ were defined. These moduli, calculated from the elastic coefficients, served as markers for the elasticity at initial (subscript _M_: minimal) and peak (_L_: large) strains. These should have been approaching the linear elastic modulus (G′_1_) obtained in small amplitude oscillatory shear at small or moderate strain levels. Complementarily, the dynamic viscosities at the onset of strain (η′M) and at its highest rate (η′L) were calculated from the corresponding viscous coefficients *v_n_*.

The elastic and the viscous Lissajous–Bowditch curves provided a geometric perspective of the viscoelastic properties for a specific strain. The inclination of the initial tangent line (zero strain) on the elastic curve corresponded to the minimum-strain modulus whereas the secant’s slope at peak strain correlated with the large-strain modulus and viscosity (Figure 5).

**Figure 6:**
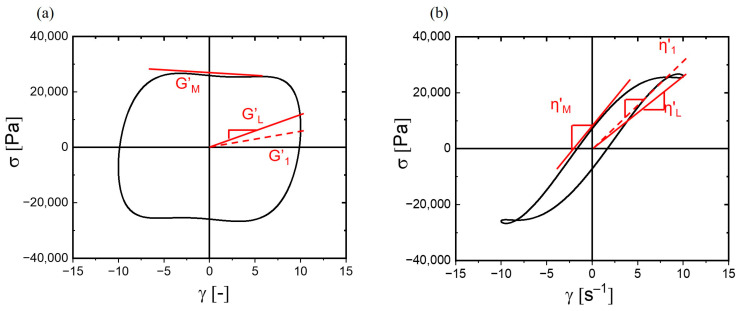
(**a**) Nonlinear elastic moduli (G′_L_ and G′_M_) and (**b**) dynamic viscosities (η′L and η′M) for a hydrogel comprising 0.5 wt% of guar at ω = 1 rad/s and γ_0_ = 1000%, T = 25 °C. Dashed lines denote the associated linear (first harmonic) material properties $G_{1}^{\prime }$ and $\eta_{1}^{\prime }$.

The collection of Lissajous–Bowditch curves into a (ω, γ_0_)-Pipkin diagram provided an initial understanding of the transition to nonlinear flow characteristics (Figure 6). This transition was marked by a deviation from the linearity of the slopes (red lines in Figure 6) of the decomposed elastic G′ resp. of the viscous η′ (= G″/ω) components, i.e., the deviations from the curves’ initially ellipsoidal shape in the linear response region of strain. The linear viscoelastic behavior, e.g., for the hydrogel based on 0.5 wt% of guar, was evident through the linear paths of both elastic and viscous stresses at strain amplitudes smaller than 10%.

**Figure 7:**
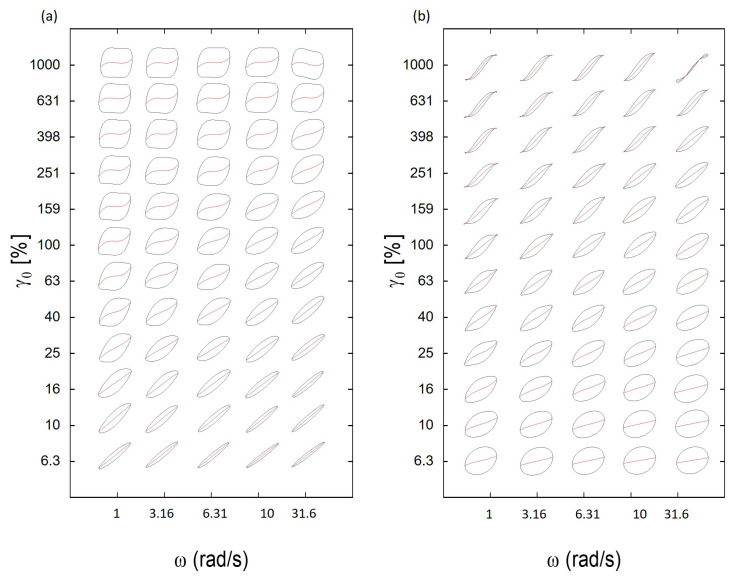
(**a**) Elastic Lissajous–Bowditch curves for hydrogel containing 0.5 wt% guar at 25 °C yielding the (ω, γ_0_)-Pipkin space, with normalized total stress σ(t)/σ_max_ (in black) and normalized elastic stress σ′(t)/σ_max_ (in red) relative to the normalized strain γ(t)/γ_0_; (**b**) corresponding viscous Lissajous–Bowditch curves with normalized total stress σ(t)/σ_max_ (in black) and normalized viscous stress σ′(t)/σ_max_ (in red) plotted against the normalized strain rate $\dot{\gamma }\left(t\right)/{\dot{\gamma }}_{0}$.

A shift in the elastic components at strains higher than 40% towards a quadrilateral formation (Figure 7) was readily detected. At larger strain, this quadrilateral form became more pronounced, consistent with the terminal slope changes in the G′ curve, signifying the limitations of the network of crosslinks and crystallites to extend beyond a certain arc length. This was further collaborated by the convexity in the decomposed elastic stress curves at higher strains. At peak strain, the elastic modulus G′_L_ surpassed G′_M_, possibly when the characteristics of the more permanent network of chemical crosslinks became dominant.

**Figure 8:**
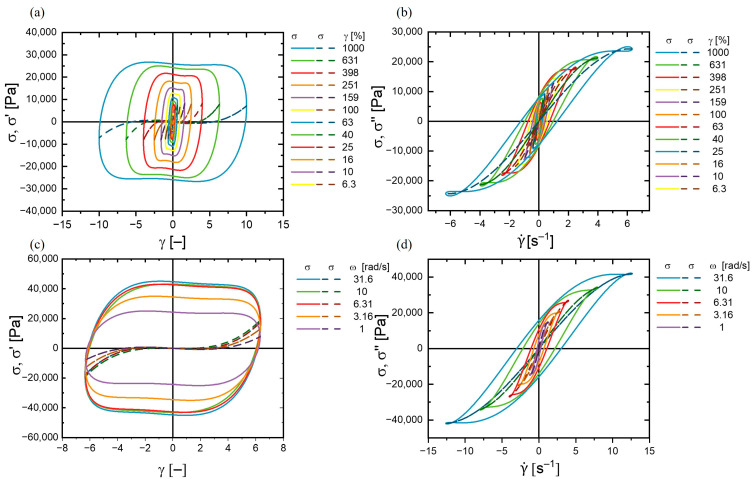
(**a**) Elastic Lissajous–Bowditch curves: (**a**) (the total stress σ(t)and the elastic stress σ′(t); viscous Lissajous–Bowditch curves: (**b**) (the total stress σ(t) and the viscous stress σ″(t) at a constant strain γ_0_ of 40%. Elastic Lissajous–Bowditch curves: (**c**) (the total stress σ(t) and the elastic stress σ′(t); viscous Lissajous–Bowditch curves: (**d**) (the total stress σ(t)and the viscous stress σ″(t) as function of the strain γ(t)) at a constant angular frequency ω of 1 rad/s.

The smaller slope of the viscous stress within the viscous Lissajous–Bowditch curves at higher frequencies at a given amplitude indicated the system’s characteristic shear thinning behavior (Figure 8). The internal structure was partly lost by the straining, allowing the system to flow more easily. This may have been related to the dynamics of the hydrogen bonds. Their time constant of the network was presumed to be lower than of the agitation, leading to a loss of interactions. Indeed, the relative shear thinning from 1 rad/s to 31.6 rad/s was smaller at higher strains, illustrating that also a larger strain of low frequency led to structural changes (Table S3). The dominance of viscous nonlinearity was particularly evident in the total stress loop’s alignment closer to the viscous stress component, especially at lower frequencies and higher strain amplitudes.

#### 3.3.4. Nonlinear Viscoelastic Parameters of Hydrogels

**Figure 9:**
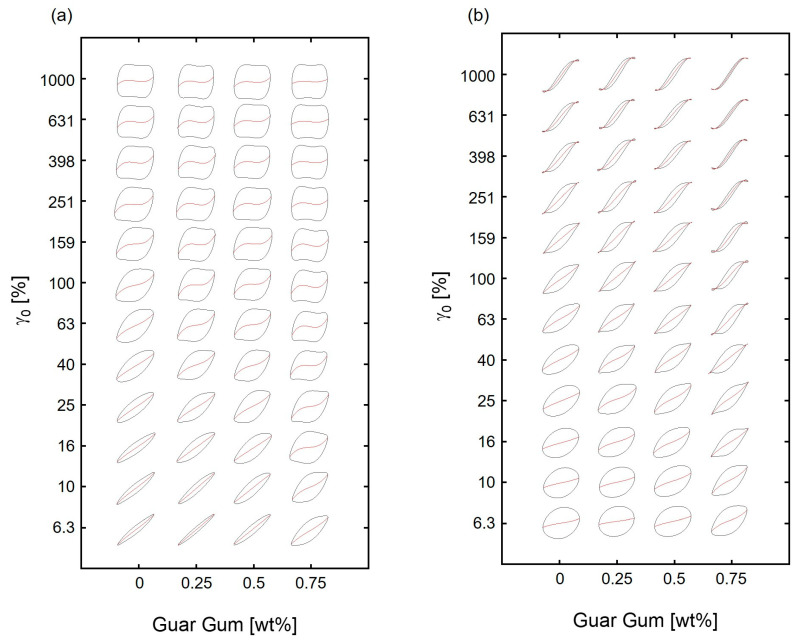
(**a**) Elastic Lissajous–Bowditch plots illustrating normalized total stress σ(t)/σ_max_ (black lines) and normalized elastic stress σ′(t)/σ_max_ (red lines) relative to normalized strain γ(t)/γ and (**b**) viscous Lissajous–Bowditch graphs displaying normalized total stress σ(t)/σ_max_ (black lines) and normalized viscous stress σ′(t)/σ_max_ (red lines) as functions of normalized strain rate $\dot{\gamma }\left(t\right)/{\dot{\gamma }}_{0}$ across varying concentrations of guar (0 to 0.75 wt%) at ω of 1 rad/s, T = 25 °C.

Guar’s influence on the hydrogels earlier was interpreted as the result of polyvinyl alcohol (PVA) crystallization, culminating in a denser hydrogel network at higher concentrations. This should have resulted in smaller network arcs with a lower mobility. Indeed, it was found that gels with a higher content of guar entered the nonlinear regime at lower strains. A comparative analysis of hydrogel samples revealed distinct nonlinear viscoelastic characteristics attributable to guar (at a shear rate of 1 rad/s; Figure 9).

Elastic curves illuminated that an increase in guar concentration results in the onset of nonlinear behavior at lower strain. A marked increase in viscous stress alongside guar concentration increments underscored its individual thickening process, which was more pronounced at higher guar concentrations (i.e., relative to the situation at rest). This earlier entrance into nonlinear viscous properties with an observed shift towards earlier quadrilateral transformations at higher guar levels aligned with previously noted reductions in both critical strain (γ_c_) and yield strain (γ_y_). A pronounced elastoplastic response emerged with guar content, signifying enhanced yield stress and, by extension, superior resistance to deformation.

**Figure 10:**
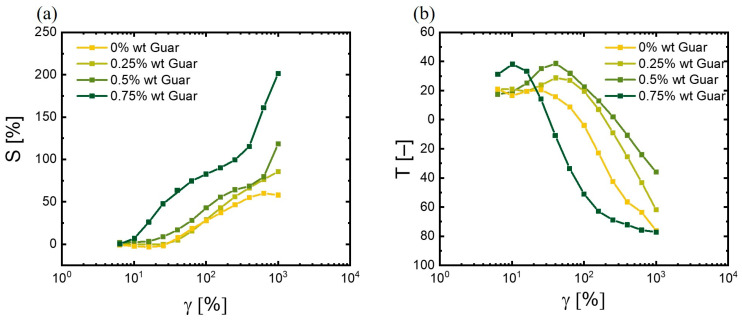
(**a**) Strain-stiffening ratio S (defined as (G′_L_ − G′_M_)/G′_L_) against strain amplitude γ_0_ and (**b**) shear-thickening ratio T (calculated as (η′L − η′M)/η′L) as functions of strain amplitude γ_0_ for guar hydrogels (ω = 1 rad/s and T = 25 °C) (C.f. Figure S6).

A quantitative analysis of the primary sources contributing to nonlinear behavior could be obtained from the intracycle nonlinear parameters (Figure 10) derived from the Lissajous plots. The strain-stiffening ratio S $\left(={\;G\prime }_{L}-{G\prime }_{M}\right)$ as the difference between large and minimum elastic moduli increased sharply with an increasing strain amplitude, being indicative of the non-Gaussian stretching of the network elements. The increase was most prominent at a higher guar concentration, consistent with a denser network of elastically active arcs.

The intracycle shear thickening in terms of the ratio T (=η′L − η′M)/η′L) ran through a (usual) maximum for all the hydrogels of this study. The decline at higher amplitudes indicated the breakdown of the physical network of the crystallites and the crosslinks (T < 0). The thinning behavior in terms of T started at strain amplitudes at around 25% for the hydrogel devoid of guar without much difference on the way. The structure and the dynamics of the gel were lost at higher strains. In contrast, the gel based on 0.75 wt% of guar had a clearer maximum, showing the higher resistance to flow as strain increased. It also had the maximum of T at a lowest strain of about 10% from whereon the internal structure started to breakdown. This paralleled the fracture characteristics of this inferior material (vide supra). The largest increase in the viscosity in the cycle of 1 rad/s was found for the gels comprising 0.25 and, in particular, 0.5 wt% of guar (rising to 25% and 40%, respectively), both at a strain of about 30%. This pointed towards a shear-induced temporary structural formation and the fast re-establishment of physical bonds disrupted during oscillatory flows. This temporary structuring may have resulted from hydrogen bonding, facilitated by shear-aligned molecular structures during deformation. The presence of more bonding sites reduced the relaxation time of chains at high strains and frequencies.

#### 3.3.5. Self-Healing in Dynamic Oscillation Experiments

**Figure 11:**
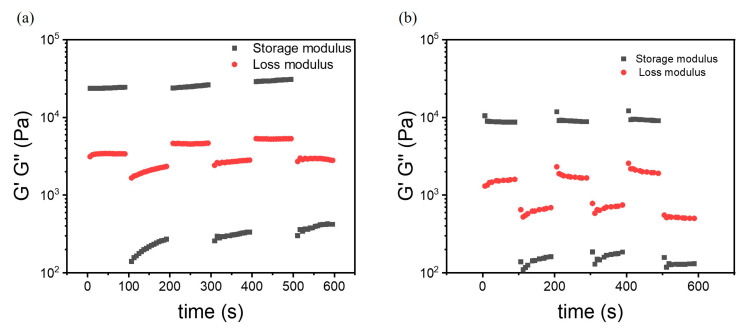
Alternating step strain sweep conducted over 100 s, cycling between 1% and 250% strain at ambient temperature with a frequency of 1 Hz on hydrogels with (**a**) 0.5 wt% and (**b**) 0 wt% of guar [15].

The optimal concentration of guar for enhancing the gel’s ability to dissipate energy seemed to lie close to 0.5 wt%. This gel appeared to be of most interest for application as a smart material with a response and a self-healing potential and was therefore tested by application of alternating levels of straining (1% and 250% stretches). It was observed that the material could switch between a fluid-like state and a gel with the amount of stretching, as the rheological LAOS characterization implied (Figure 11). At a lower stretch, the hydrogel was dominantly elastic, with its ability to store energy being much higher than its ability to dissipate energy. However, applying a larger stretch resulted in the transitioning from a near-solid to a near-liquid state, with an increase in energy dissipation indicating that the gel’s network was partly breaking down. After returning to the lower strain, the hydrogel’s elastic properties were not only recovered but slightly enhanced. The main change took place in the second part of the first cycle, after which the gel became more consistent. This cycle of applying and releasing strain could be repeated multiple times, each time slightly increasing the hydrogel’s storage and loss moduli from their original values. This suggested that the hydrogel with guar adapted to the stretching and compressing, potentially becoming more elastic over time. The values of the moduli of the gel were appreciably higher than those of the gel without guar (Figure 11b), showing the improvement of the addition of guar.

The hydrogel’s ability to return to its original state after being stretched indicated its thixotropic nature to lose much of its internal (H-bonding) structure under stress and to recover its solidity when the stress was removed. This recovery was attributed to the mainly physical makeup of the hydrogel, particularly the guar and TA, which formed a network that provided the gel with self-healing and a “memory” of its original shape. This behavior pointed to the complex interactions within the hydrogel that contributed to its elasticity and its abilities to withstand deformation and recover efficiently.

### 3.4. Thermo-Responsive Behavior

**Figure 12:**
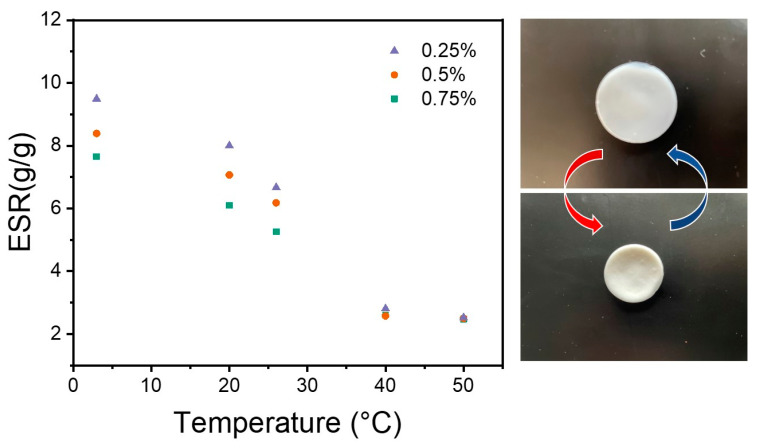
Equilibrium swelling ratio (ESR) with temperature for the guar-based hydrogels and the picture of 0.5 wt% guar hydrogel showing swelling and deswelling status.

The hydrogels underwent the anticipated phase transition around 30 °C, aligning with the thermo-responsive behavior expected from NIPAAM-based polymers, which displayed a lower critical solution temperature (LCST) in that range. A key observation was the reduction in the swelling ratio as the concentration of guar increased (Figure 12). This went along with the crystallinity of the samples, with the crystalline parts not taking up much water. The strongest hydrogel with 0.5 wt% guar still had a substantial swelling ratio change from 8.4 at 5 °C to 2.5 at 50 °C, showcasing its effective water absorption and release property.

#### Dynamic Thermo-Responsiveness Properties

**Figure 13:**
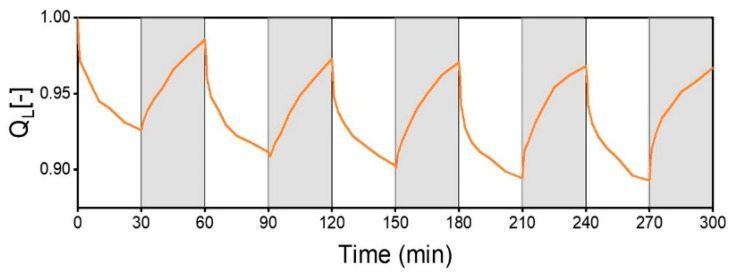
Dynamic swelling degree normalized by length (Q_L_) for the hydrogel with 0.5 wt% of guar under cyclic temperature alternation between 40 °C and ambient conditions (grey shading) in deionized water.

The dynamic response to temperature variations was monitored through five alternating cycles of swelling at 40 °C and deswelling at 25 °C with 0.5 wt% of guar (Figure 12). The hydrogel exhibited a rapid thermo-response to temperature changes, particularly during the deswelling phase, where a significant volume reduction occurred within the first five minutes. This phase was followed by a markedly slower process (Figure 13). The rehydration phase at 25 °C had a similar complex behavior, initially showing a quick expansion before transitioning to a more gradual increase. This multi-phase kinetic pattern is a general feature of PVA/PVA-MA/TA hydrogels [15]. The additional presence of guar tended to smooth the transition between the fast and slower phases.

**Figure 14:**
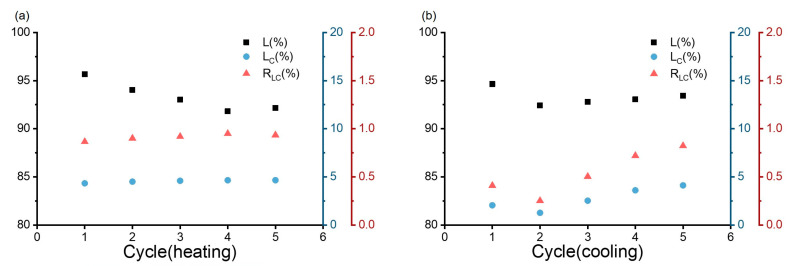
Measurements of length (L), ultimate length alteration (L_C_), and the corresponding linear rate constant in %/min (R_LC_) for guar hydrogel in deionized water during cycling of 30 min intervals between 40 °C (**a**) and 20 °C (**b**).

Again it was observed that the maximum diameter of the gel diminished with the cycle count but, advantageously, only in the initial three cycles. Thereafter, a more constant volume was reached, which was important for application as a valve in a smart reactor [32]. A similar observation was made for the initial rate constants of swelling and deswelling (Figure 14); they favorably increased with the cycle count. Apparently, the amount of water contained in the gels after the synthesis was higher than the amount involved in swelling–deswelling cycles. It may have been anticipated that the loss of water led to the establishment of new hydrogen bonds between guar, TA, and PVA. These may have significantly enhanced the hydrogel’s restructuring capacity. The introduction of guar with its high molecular weight of over 1 × 10^6^ Da may have additionally established a longer-range connectivity in the amorphous, swelling part, linking itself also to TA and PVA by hydrogen bonding and also by integration into the PVA-MA network by the radical polymerization. Both guar and TA would contribute to hydrogel’s self-healing features with the conservation of its external form. The resultant network architecture would ensure the hydrogel to maintain more uniform dimensions in both its swollen and contracted states, underscoring its remarkable dimensional stability.

The initial phase of the deswelling behavior of the 0.5 wt% guar hydrogel (Figure 13) could reasonably be described as an exponential decay (Figure S7a). A rate constant for this phase of just smaller than 0.01 s^−1^ could be extracted. This seemed to hold true until a compressional strain of about 0.75. The deswelling went along with water being expelled, driven by the generation of new (hydrogen) bonds between the components of the hydrogel. Taking the compression and tensile stress for the smaller changes of 25% in dimension equal, the initial stress to be overcome by the formation of new bonds was about 6 kPa (extrapolated from the total stress at ω=1 rad/s; Figure S7). The gel progressively changed on deswelling as more polymer–polymer interactions formed. This structural change would enhance the elastic modulus and the total stress in the network at further compression increased. This may already have been indicated by the strain stiffening ratio S and the thickening ratio T. Also, the rate of change decreased. The slower deswelling phase corresponded to the reorganization of the network and the reformation of transient hydrogen bonds. Studies on the gels at temperatures close to the LCST are planned to address the mechanics of the gels in the transition to the de-swollen network.

## 4. Concluding Remarks

Guar’s impact on the PVA/TA/PVA-MA-g-PNIPAAm hydrogel’s structural, mechanical, and viscoelastic properties arises from its ability to modulate PVA crystallization, alter the polymer network architecture, and contribute to dynamic physical interactions. Guar incorporation increased the hydrogel’s crystallinity from 14.6% (no guar) to 24.2% and 30% at concentrations of 0.5 wt% and 0.75 wt%, resulting in finer and more PVA crystallites. At 0.5 wt%, the hydrogel exhibited superior tensile strength (620 kPa) and elongation at break (500%); the mechanical robustness at this concentration was attributed to a balanced interplay between enhanced crystallinity and the formation of transient hydrogen-bonding interactions between guar, tannic acid (TA), and PVA. The same way, in its viscoelastic behavior, guar enhanced the strain hardening effect of the hydrogel, with the third harmonic (I_3_/I_1_) slope reducing from 1.46 (no guar) to 0.744 (0.75 wt% guar), reflecting the formation of denser and more entangled polymer networks. The shear-thickening ratio increased by 40% in the formulation with 0.5 wt% guar, indicating improved network connectivity and energy dissipation under high strain; higher guar concentrations (0.75 wt%) conversely led to early structural breakdown under strain, marked by a reduction in yield strain (γ_y_) from 46% (no guar) to 11.7%. The inclusion of guar significantly improved dimensional stability, mitigating the progressive shrinkage observed in previous PVA/TA-based hydrogels. The thermal responsiveness and swelling properties of the 0.5 wt% guar hydrogel showed a substantial reduction in swelling capacity (with the ESR changing from 8.4 at 5 °C to 2.5 at 50 °C) while preserving superior dimensional stability across multiple swelling–deswelling cycles, confirming its improved long-term performance. This study provided a comprehensive understanding of the structure–property relationships within guar-modified TN hydrogels and underscored guar’s potential in tailoring the mechanical behavior of advanced hydrogel systems.

## Figures and Tables

**Table 1:** Physical properties of PVA/TA/PVA-MA-g-PNIPAAm guar hydrogels.

Guar Content (wt%)	0	0.25	0.50	0.75
XRD (peak at 19.6°)				
Integral value	67,688	128,415	129,203	140,184
Peak	6049	15,057	15,907	17,406
FWHM * (°)	15.1	8.12	7.76	7.66
DSC				
Tm (°C)	221.8	225	223.4	225.5
Melting range (°C)		213.7–230.4	212.6–229.7	214.9–230.3
ΔH_m_ (J/g)	20.1 ± 0.1	30.1 ± 0.9	33.9 ± 0.4	41.2 ± 0.5
Crystallinity (%)	14.6	22.0	24.2	30

*: Full width at half peak height.

**Table 2:** Strain-dependent rheological properties of PVA/PVA-MA/TA/guar hydrogels.

Guar Content (wt%)	0	0.25	0.5	0.75
Initial critical strain(γ_c_) %	13.7	7.2	9.6	2.8
Subsequent yield strain (γ_y_) %	46.0	29.6	34.4	11.7
Max of G″ (strain%)	40	25	25	10
Max of G″ (kPa.s)	3.3	5.5	11.4	4.3

## Data Availability

The original contributions presented in this study are included in the article. Further inquiries can be directed to the corresponding author.

## Supplementary Materials

The following supporting information can be downloaded at: https://www.mdpi.com/article/10.3390/polym17050597/s1, Figure S1: IR spectra of the guar gum solution, hydrogel precursor, and fabricated hydrogel of 0.5 wt% guar gum; Figure S2: XRD spectra of the hydrogel. with 0.25 wt%. 0.5 wt%. and 0.75 wt% guar gum; Table S1: Mechanical data of hydrogels of different guar contents; Figure S3: The amplitude sweeps (a) and the frequency sweeps (b) of the hydrogels with 0.5 wt% guar gum; Table S2: Entanglement data of hydrogel at 25 °C; Figure S4: The square of average molecular weight of the effective crosslinks (entanglements) as function of the strain at break (λ_max_) of hydrogels with different contents of guar; Figure S5: The storage modulus G′ and the loss modulus G″ as functions of the strain amplitude γ_0_ for hydrogel with 0.5 wt% guar gum at ω = 1 rad/s and the corresponding stress response in SAOS and LAOS; Table S3: Slopes of normalized viscous stress of hydrogel with 0.5 wt% guar; Figure S6: Minimum-strain elastic modulus G′_M_, large-strain elastic modulus G′_L_, and linear (first-harmonic) elastic modulus G′_1_ against strain amplitude γ^0^; (b) minimum-strain viscosity η′M, large-strain viscosity η′L, and linear (first-harmonic) viscosity η′1 as functions of strain amplitude γ^0^ for guar hydrogels (ω = 1 rad/s and T = 25 °C); Table S4: Equilibrium swelling ratio (ESR) of different contents of guar hydrogel; Figure S7: The fourth loop of the deswelling curve and the corresponding paragraph (a), the data of the total stress and viscous stress of the same shear rate extracted from the LAOS result (b,c), and the total stress and viscous stress change with respect to time in the deswelling (d).
