# Supplementary material for: Nonlinear Viscoelasticity of and Structural Modulation in Guar Gum-Enhanced Triple-Network Hydrogels
Source: Polymers (Basel). 2025 Feb 24;17(5):597. doi: 10.3390/polym17050597 (PMC11902840; doi:10.3390/polym17050597)
Supplement: Supplementary file 1 [file polymers-17-00597-s001.zip › polymers-3478180-supplementary.pdf]

Supplementary Materials

Nonlinear Viscoelasticity of and Structural Modulation in Guar Gum-Enhanced Triple-Network Hydrogels

Yi Luo [1], Werner Pauer [2] and Gerrit A. Luinstra [2,*]

* Correspondence: luinstra@chemie.uni-hamburg.de

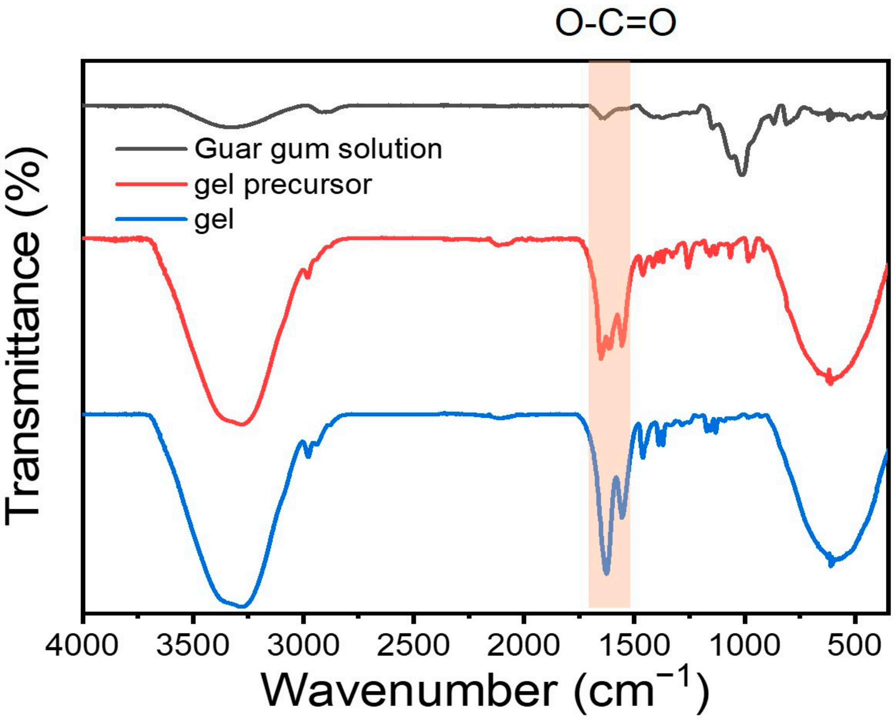

Figure S1. IR spectra of the Guar gum solution. hydrogel precursor. and fabricated hydrogel of 0.5 wt.% Guar gum.

Academic Editor: Firstname Lastname

Received: date
Revised: date
Accepted: date
Published: date

Citation: To be added by editorial staff during production.

Copyright: © 2025 by the authors. Submitted for possible open access publication under the terms and conditions of the Creative Commons Attribution (CC BY) license (https://creativecommons.org/licenses/by/4.0/).

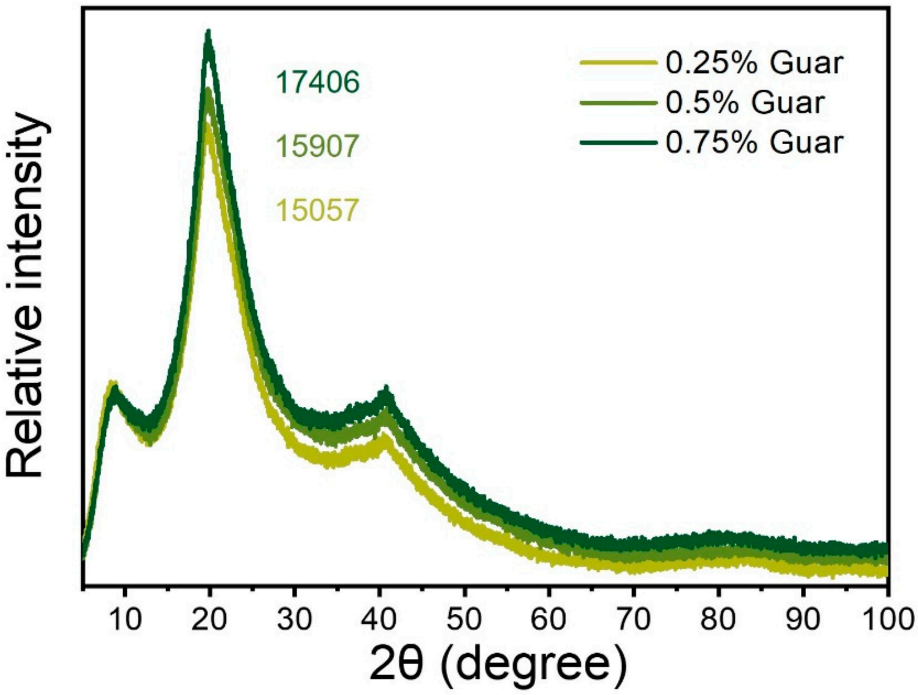

Figure S2. XRD spectra of the hydrogel. with 0.25 wt.%. 0.5 wt.%. and 0.75 wt.% Guar gum.

Table S1. Mechanical data of hydrogels of different guar content.

Guar content of hydrogel (wt%)	Rubbery plateau modulus (G') (Pa)	Chain length between crosslinks ($\overline{M_e}$) (g/mol)	The strain at break(λ_{max})
0	11214.4	276.16	7.04
0.25	17964.7	172.39	4.05
0.5	29947.2	103.41	5.04
0.75	13410.4	230.94	3.25

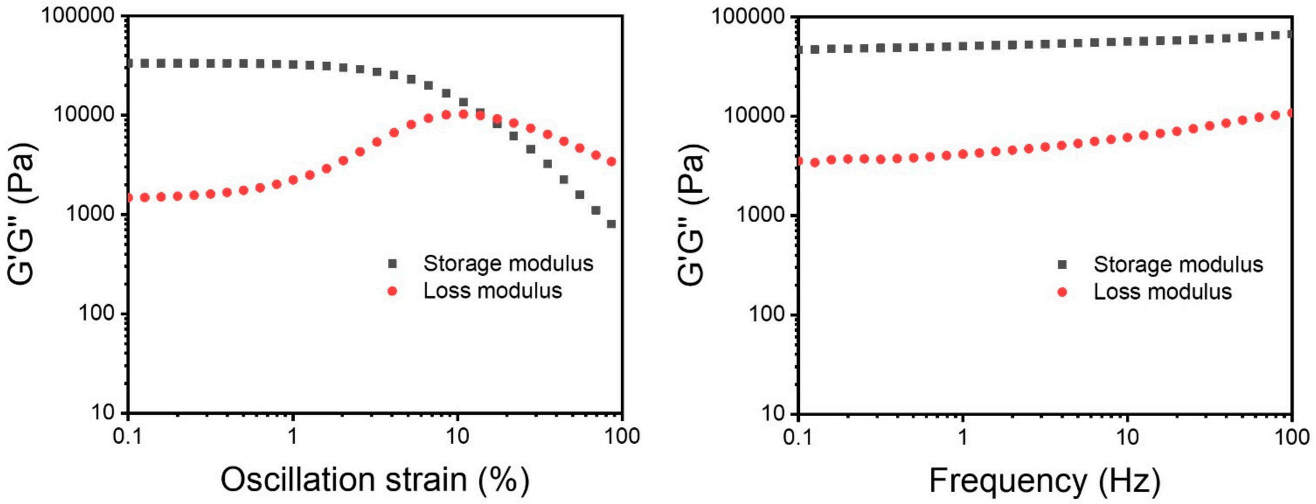

Figure S3. The amplitude sweeps (a) and the frequency sweeps (b) of the hydrogels with 0.5 wt% Guar gum.

Table S2. Entanglement data of hydrogel at 25°C.

Guar content of hydrogel (wt%)	Rubbery plateau modulus (G') (Pa)	Chain length between crosslinks ($\overline{M_e}$) (g/mol)	The strain at break(λ_{max})
0	11214.4	276.16	7.04
0.25	17964.7	172.39	4.05
0.5	29947.2	103.41	5.04
0.75	13410.4	230.94	3.25

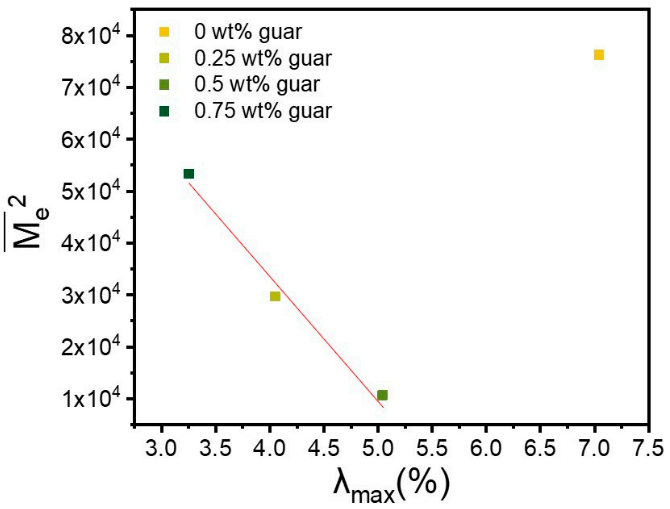

Figure S4. The square of average molecular weight between the effective crosslinks (entanglements) as function of the strain at break (λ_{max}) of hydrogels with different contents of guar.

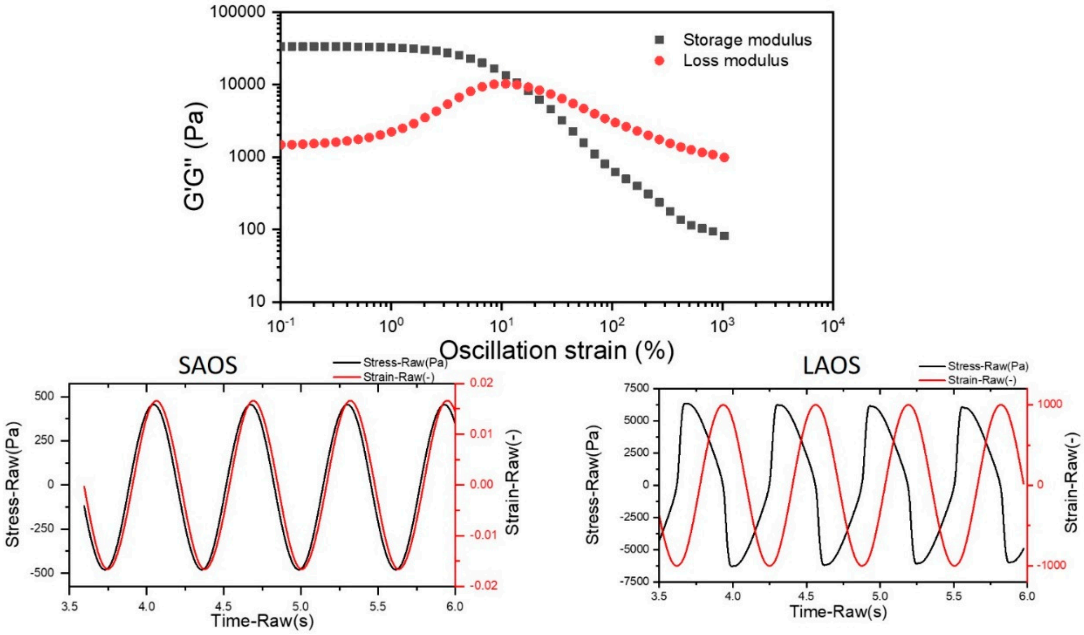

Figure S5. The storage modulus G' and the loss modulus G'' as functions of the strain amplitude γ_0 for hydrogel with 0.5 wt% Guar gum at ω =1 rad/s and the corresponding stress response in SAOS and LAOS.

Table S3. Slopes of normalized viscous stress of hydrogel with 0.5 wt% Guar.

ω (rad/s)<br>γ_0 (%)	1	3.16	6.31	10	31.6
100	91.3	90.8	80.6	72.5	53.3
60	82.5	78.2	65.4	56.2	42.6
40	64.1	62.3	50.2	43.5	31.4
25	56	48.3	38.4	34.8	27.2
16	34.6	36.4	30.6	29	24.1
10	32.7	28.7	25.8	24.9	21.8
6.3	27.5	24.3	22.6	22.1	20.1

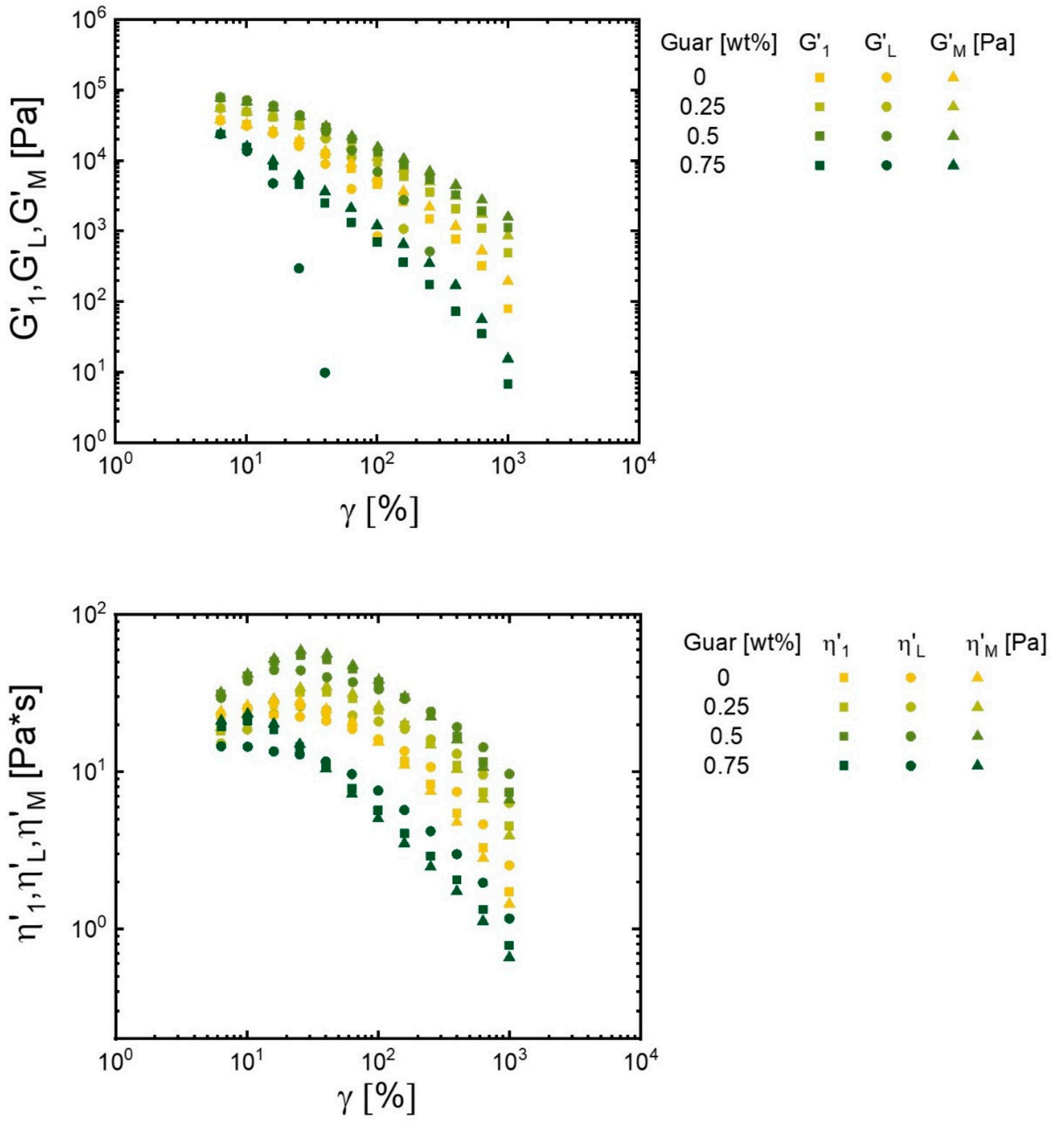

Figure S6. Minimum-strain elastic modulus G'_M. large-strain elastic modulus G'_L. and linear (first harmonic) elastic modulus G'_1 against strain amplitude γ_0. (b) minimum-strain viscosity η'_M. large-strain viscosity η'_L. and linear (first harmonic) viscosity η'_1 as functions of strain amplitude γ_0 for guar hydrogels (ω = 1 rad/s and T = 25 °C).

Table S4. Equilibrium swelling ration (ESR) of different contents of guar hydrogel.

Guar content(wt%) / Temperature (°C)	0.25	0.5	0.75
3	9.49	8.39	7.65
20	8	7.07	6.1
26	6.67	6.18	5.26
40	2.81	2.58	2.61
50	2.52	2.5	2.48

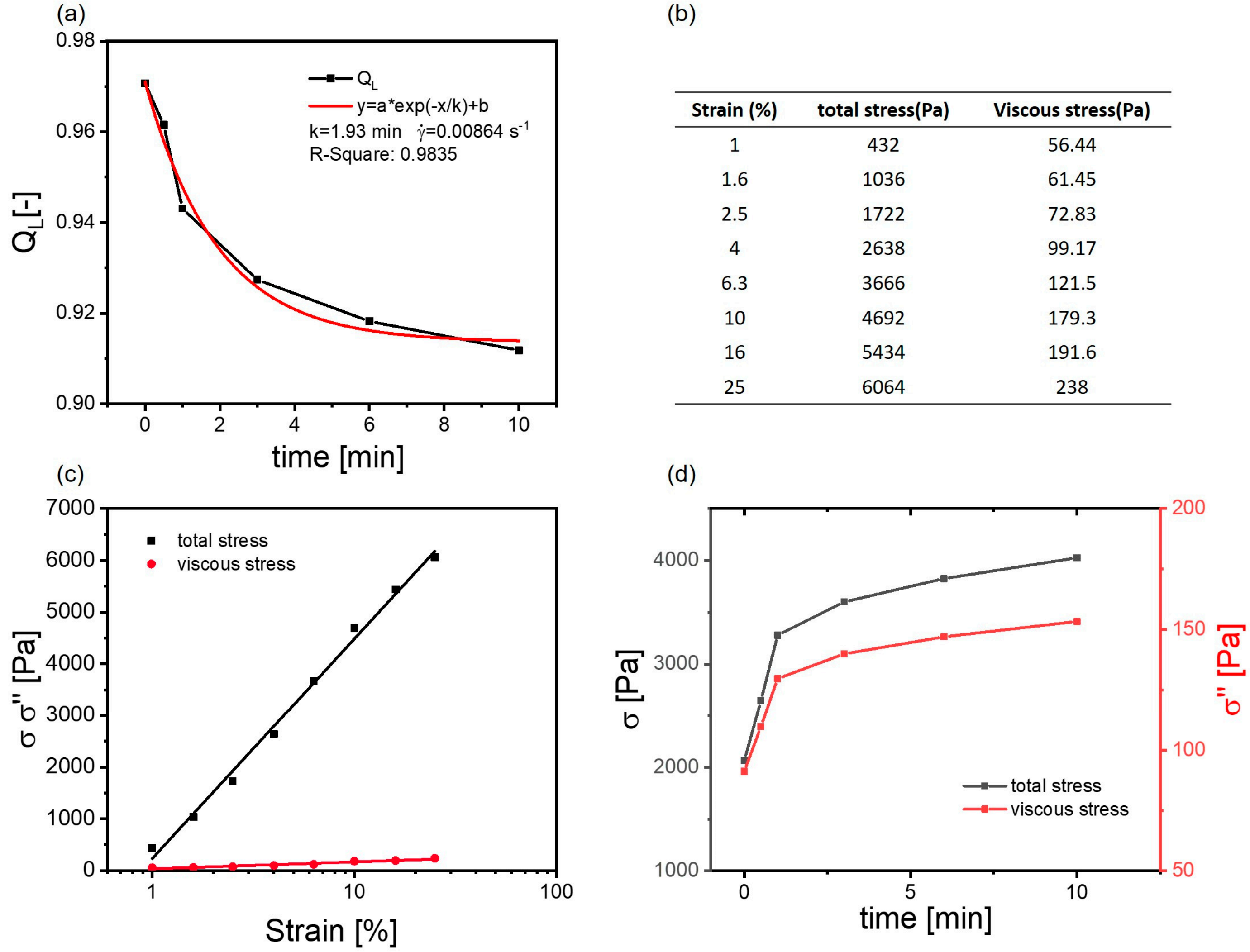

Strain (%)	total stress(Pa)	Viscous stress(Pa)
1	432	56.44
1.6	1036	61.45
2.5	1722	72.83
4	2638	99.17
6.3	3666	121.5
10	4692	179.3
16	5434	191.6
25	6064	238

Figure S7. The fourth loop of the deswelling curve and the corresponding paragraph (a), the data of the total stress and viscous stress of the same shear rate extracted from the LAOS result (b, c), the total stress and viscous stress change with respect to time in the deswelling(d).
